# Short-term efficacy of the IL6 receptor antibody tocilizumab in patients with HIV-associated multicentric Castleman disease: report of two cases

**DOI:** 10.1186/1756-8722-7-10

**Published:** 2014-01-17

**Authors:** Azusa Nagao, Shoko Nakazawa, Hideji Hanabusa

**Affiliations:** 1 Ogikubo hospital, Imagawa3-1-24, Suginami-ku, Tokyo 167-0035, Japan

**Keywords:** Castleman disease, HHV8, IL6, HIV-MCD, Tocilizumab

## Abstract

Multicentric Castleman disease (MCD) is a lymphoproliferative disorder caused by human herpesvirus 8 (HHV8) infection HIV associated MCD (HIV-MCD) presents with various clinical symptoms. Many HIV-negative MCD patients are often treated with anti-human interleukin-6 (IL6) receptor monoclonal antibodies (tocilizumab), and successful results have been reported. IL-6 plays an important role in the development of both HIV-positive and HIV-negative MCD; however, the efficacy of tocilizumab in HIV-MCD patients is unknown. We herein report the clinical and biologic courses of two HIV-MCD patients treated with tocilizumab. In both cases, a significant and rapid clinical improvement was observed after the first infusion. However, the treatment efficacy was not maintained for a long period, and relapse occurred at 15 and 22 weeks, respectively. Both patients received rituximab and subsequently achieved complete clinical remission. Our report, in addition to data presented in the literature, suggests that tocilizumab could be an initial treatment option in patients with HIV-MCD.

## Background

Multicentric Castleman disease (MCD) is a lymphoproliferative disorde, and HIV-associated MCD (HIV-MCD) is caused by human herpesvirus 8 (HHV8) infection in HIV-positive patients [1]. HIV-MCD presents with various clinical symptoms, including fever, swelling of the spleen, liver and systemic lymph nodes and abnormalities in laboratory values, such as findings of anemia, thrombocytopenia or hypergamma-globulinemia, as well as a low albumin, or high C-reactive protein (CRP) level. HHV8 resides latent infection and replicates in the plasmablasts of lymph nodes under conditions of immunodeficiency. Many HIV-negative MCD patients are treated with anti-human interleukin-6 (IL6) receptor monoclonal antibodies (tocilizumab), with successful results having been reported [2]. IL-6 plays an important role in the development of both HIV-positive MCD and HIV-negative MCD; however, the efficacy of tocilizumab in HIV-MCD patients is unknown. We herein report the results of two HIV-MCD patients treated with tocilizumab.

### Case presentation

#### ***Case 1***

A 44-year-old male who was HIV-1 seropositive for several years and did not start treatment with combination antiretroviral therapy (cART), with a CD4 cell count of 188 cells/μl and a viral load of 74 copies/μl, was diagnosed with Kaposi’s sarcoma and treated with two cycles of liposomal doxorubicin and cART. Hepatitis C and B were negative. Eight months after being diagnosed with Kaposi’s sarcoma, he presented with a high fever, fatigue and lymph nodes swelling throughout his body. Blood tests revealed anemia (hemoglobin: 8.3 g/dl), thrombocytopenia (3.3×104/μ), a low albumin level (2.3 g/dl) and a high CRP level (10.75 mg/dl). The high fever persisted for two weeks. A lymph node biopsy demonstrated remarkable infiltration of polyclonal plasma cells and plasmablastic cells in the interfollicular areas. Lymph node architecture was retained. Vascular proliferation was observed between the follicles, with perivascular hyalinization. The levels of HHV8 and human IL6 (hIL6: reference normal value <4.0 pg/mL) in the blood were 460,000 copies/μl and 41.7 pg/ml, respectively. The patient was diagnosed with HIV-MCD and 8 mg/kg of tocilizumab was administered intravenously. The persistent high fever disappeared within a few hours. There were no adverse events of tocilizumab treatment. After one week, the laboratory abnormalities recovered: hemoglobin 10.8 g/dl, platelets 11.2×10^4^/μ, albumin 3.8 g/dl and CRP 0.15 mg/dl.

**Figure 1:**
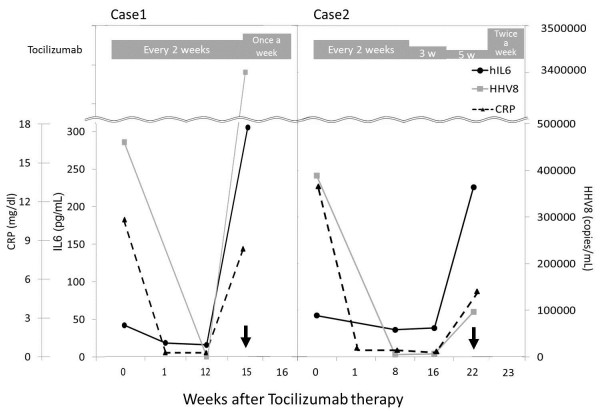
**hIL6, HHV8 and CRP dynamic.** Changes in the levels of human interleukin-6 (hIL6), human herpesvirus 8 (HHV8) DNA and serum C-reactive protein (CRP) in Cases 1 and 2 following the initiation of tocilizumab therapy. ● hIL6, △CRP and ■ HHV8 in the serum. ↓ The arrows indicate the time of relapse. The gray boxes indicate the frequency of tocilizumab infusion.

The HHV8 concentration and hIL6 level in the blood decreased to 120 copies/μl and 18.2 pg/ml, respectively, after treatment (Figure 1, Case 1). Treatment with tocilizumab was continued once every two weeks, and the patient remained symptom-free for eight cycles. However, 15 weeks after the start of treatment, symptom relapse occurred, with a high fever, fatigue and lymph nodes swelling. The CD4 count had increased from 150 to 250 cells/μl; however, at the time of relapse, the CD4 count was 109 cells/μl. Blood tests showed a hemoglobin level of 7.7 g/dl, a platelet count of 4.3×10^4^/μ, an albumin level of 2.1 g/dl, a CRP level of 8.18 mg/dl, an HHV8 titer of 3,400,000 copies/μl and a hIL6 level of 305 pg/ml, indicating HIV-MCD relapse. A second lymph node biopsy showed angiofollicular hyperplasia and interfollicular plasma cell infiltration. HHV8 antigens were more strongly positive in lymphocytes than that observed on the first biopsy. The patient received tocilizumab infusions once in week for two weeks (the 15th and 16^th^ weeks); however, his symptoms and blood test abnormalities worsened. Tocilizumab was discontinued and he recovered following the administration of four cycles of rituximab treatment. He has since remained in remission for four years.

#### ***Case 2***

A 45-year-old male with HIV infection, a CD4 cell count of 328 cells/μl and an HIV RNA level of 83 copies/μl had received cART for several years. The patient was also infected with hepatitis C virus (genotype 1b), although hepatitis B was negative. In 2012, he presented with a high fever and fatigue with swollen lymph nodes throughout his body. A blood test showed anemia (a hemoglobin level of 6.1 g/dl), a low albumin level (2.4 g/dl), a high CRP level (13.59 mg/dl), a low platelets count (8.7×10^4^/μl) and hyper gammaglobulinemia (2,993 mg/dl). He was diagnosed with HIV-MCD based on the findings of a lymph node biopsy. The follicles showed varied degrees of involution and hyalinization of the germinal centers with prominent mantle zones that intruded into the germinal centers, completely effacing them. In the mantle zone cells, variable numbers of plasma cells and lymphocytes with HHV8 antigens were observed. The levels of hIL6 and HHV8 in the blood were 53.5 pg/ml and 390,000 copies/μl, respectively. The patient received 8 mg/kg of tocilizumab 10 days after appearance of symptoms. Following the administration of tocilizumab, the high fever immediately decreased. The abnormal values on the blood examinations improved two weeks after treatment: hemoglobin 14.8 g/dl, platelets 12.9×10^4^/μl, albumin 4.4 g/dl and CRP 0.07 mg/dl. The levels of HHV8 and hIL6 decreased to 5,100 copies/μg and 35 pg/ml, respectively, eight weeks after treatment (Figure 1, Case 2). Six cycles of tocilizumab were administered once every two weeks for 10 weeks and the patient’s symptoms disappeared. The CRP level became negative (<0.3 mg/dl), and the dosing intervals were extended in a stepwise fashion to two cycles every three weeks then one cycle every five weeks. There were no adverse effects of tocilizumab treatment. However, 22 weeks after the starting of tocilizumab therapy, one week after the ninth tocilizumab infusion, a high fever, lymph node swelling and blood test abnormalities were detected. The levels of hIL6 and HHV8 were extremely high, at 219 pg/mL and 97,000 copies/ul, respectively. The CD4 count was 300–350 cells/μl, although it decreased to 164 cells/μl at recurrence. Two additional tocilizumab injections within one week (the 23rd week) were ineffective. The patient subsequently received rituximab for four cycles, after which all of his symptoms and outlier values recovered. He has exhibited no relapse for the last year.

## Discussion

In this study, infusion of tocilizumab resulted in the rapid improvement of the patients’ symptoms with no adverse effects, in addition to normalization of their blood abnormalities associated with HIV-MCD. However, the response was of short duration in both cases after the end of therapy.

HIV-negative MCD patients treated with tocilizumab exhibit very higher levels of hIL6 after treatment because hIL6 receptors are blocked by IL-6 receptor antibodies [2]. However, in the present cases, the hIL6 and HHV8 levels decreased after treatment. This suggests that HHV8 replication due to immunodeficiency was the primary pathogenesis of HIV-MCD in these cases, resulting in a vicious cycle of hIL6 production being blocked by the inhibition of IL6 signal transmission following the administration of tocilizumab. HIV-negative MCD patients were successfully treated with tocilizumab in two open label trials. However, Nisimoto et al. reported that only 2/28 patients were seropositive for HHV8 [2]. The pathophysiology of HIV-MCD may differ from that of HIV-negative MCD. HHV8 encodes viral IL6 (vIL6), a homolog of hIL6. The amino acid sequence of the vIL6 protein is 24.7% identical to hIL6 [3]. Because vIL6 stimulates HHV 8 replication and hIL6 secretion in a direct autocrine manner to promote the proliferation and survival of cells in which HHV8 are produced or in a paracrine manner to induce hIL6 in surrounding cells, it can also increase the level of hIL6 [1,4]. Tocilizumab cannot directly inhibit vIL6 receptors or vIL6 production induced by HHV8. The amount of vIL6 production is low, and the binding affinity of vIL6 to human IL6 receptors is weak. The present cases suggest that an increased serum concentration of hIL6 produced by HHV8-positive cells plays a more important role in the pathogenesis of HIV-MCD than vIL6 in the initial phase, as tocilizumab was effective in both cases. In experiments of mice, transfer of the vIL-6 transgene onto an IL-6 deficient genetic background abrogated MCD-like phenotypes, indicating that endogenous IL-6 is a crucial cofactor in the natural history of the disease [4]. Moreover, with respect to clinical data, Polizzotto M. et al. reported that, in their study, the IL-6 profiles of MCD patients fell into three distinct categories: elevation of vIL6 only (6%), elevation of hIL-6 only (50%) and elevation of both hIL-6 and vIL-6 (38%). The patients with flares associated with both elevated hIL-6 and vIL-6 levels exhibited worse symptoms [5]. The inhibition of hIL6 by tocilizumab reduces the replication of HHV8 in B-cells and decreases the hIL6 levels in the blood. An increased hIL-6 level stimulates HHV8 replication, resulting in higher levels of both vIL6 and hIL6. Tocilizumab may break this vicious cycle by blocking hIL-6 at initial phase and consequently switching off HHV8 replication in B cell. However, tocilizumab, by unknown mechanisms was ineffective at the relapse during continued treatment. Because the CD4 count was low in both patients at the time of relapse in spite of undetectable HIV RNA levels in the serum, the occurrence of HIV-MCD flares may be dependent on HIV-induced immunodeficiency, and controlling HIV infection is a major factor in patients with HIV-MCD.

Rituximab therapy is able to suppress the symptoms and signs of inflammation and decrease the risk of lymphoma in patients with HIV-MCD [6-8]. Gérard L. et al. reported that, in their studies, the three-month response rate to rituximab was 78.3% in MCD and the two-year MCD flare-free survival was 74.7% [7]. However, in these studies, Kaposi’s sarcoma flares were observed in eight of 12 patients and four of 11 patients, respectively [6,8]. In the present study, Kaposi’s sarcoma reactivation was not observed under tocilizumab and rituximab therapy in Case 1.

It is not known how much or how often tocilizumab should be administered in HIV-MCD patients or whether combination therapy with rituximab and tocilizumab is better than monotherapy with rituximab or ganciclovir. Although the effects of tocilizumab have been studied more extensively in HIV-negative patients with the refractory form of MCD, our HIV-positive patients did not respond to tocilizumab in the remission phase. We propose the use of tocilizumab treatment in patients with HIV-positive MCD as first-line therapy. It is very important to elucidate the precise pathophysiology of HIV MCD in order to select appropriate therapeutic medications. Clinical studies of tocilizumab, used alone and in combination with AZT and/or valganciclovir, are underway (NCT01441063) and will assist in elucidating these issues. The accumulation and analysis of case reports is required to evaluate when and how tocilizumab should be administered with or without other drugs, including rituximab, ganciclovir and etoposide, in patients with HIV-MCD.

## Consent

Written informed consent was obtained from the patient for publication of this case report and any accompanying images. A copy of the written consent is available for review by the Editor-in-Chief of this journal.

## Abbreviations

IL6: Anti-human interleukin-6; HIV-MCD: HIV associated multicentric Castleman disease; HHV8: Human herpesvirus 8; CRP: C-reactive protein; cART: Combination antiretroviral therapy; hIL6: Human IL6; vIL6: Viral IL6.

## Competing interests

Although Hanabusa H. has received honoraria from Baxter Healthcare, NovoNordisk, Bayer, Pfizer, and KaketsuKen; and has served on advisory boards for Baxter Healthcare (limited to hemophilia care), he has no competing interest related with this case report (Tocilizumab and Chugai Co.). Nagao and Nakazawa declare that they have no competing interests.

## Authors’ contributions

AN, SN and HH cared for the patients. AN wrote the manuscript. HH designed the study and helped to draft the manuscript. All authors have read and approved the final manuscript.
